# Contemporary prevalence and predictors of anxiety among patients living with HIV/AIDS in Ghana

**DOI:** 10.4314/gmj.v56i3.6

**Published:** 2022-09

**Authors:** John-Paul Omuojine, Samuel B Nguah, Nana K Ayisi-Boateng, Fred S Sarfo, Bruce Ovbiagele

**Affiliations:** 1 Kwame Nkrumah University of Science and Technology, Kumasi, Ghana; 2 Komfo Anokye Teaching Hospital, Kumasi, Ghana; 3 Department of Neurology, University of California, San Francisco, USA

**Keywords:** cART, mood disorders, Africa, HIV, depression, anxiety

## Abstract

**Objectives:**

To identify the prevalence and associated factors of anxiety in people living with HIV/AIDS in a tertiary centre in Ghana.

**Design:**

The study employed a cross-sectional design.

**Setting:**

The study was conducted in the outpatient HIV clinic of a tertiary hospital.

**Participants:**

Participants were adult PLWHA receiving OPD care, including those established on combined antiretroviral therapy (cART) and newer patients who were not on cART. Four hundred ninety-five participants aged ≥30 years were consecutively enrolled on the study.

**Interventions:**

Demographic and clinical data were collected using standard questionnaires and patient files. Anxiety was assessed using the Hospital Anxiety and Depression Scale (HADS). Multivariate logistic regression analysis was done to identify associated factors.

**Main outcome measure:**

Proportion of PLWHA who had HADS score of ≥8

**Results:**

Overall prevalence of anxiety was 61.0% (95%CI: 56.6 – 65.3), with no significant difference between recently diagnosed (≤ 6 months, 64.3%) and those with established diagnoses (>6 months, 59.1%). Urban residence (aOR: 1.67, 95%CI: 1.12 – 2.51), alcohol use (aOR: 1.64, 95%CI: 1.13 – 2.38) and depression (aOR: 13.62, 95%CI: 7.91 – 23.45) were independently associated with anxiety.

**Conclusion:**

In this sample, 6 in 10 Ghanaian PLWHA had evidence of anxiety. Liaison with the national mental health service for more comprehensive and integrated care and further research into the mental health of PLWHA is recommended to reduce this high burden of anxiety.

**Funding:**

This study was funded by a grant from the National Institutes of Health Fogarty International Center (R21 TW010479)

## Introduction

Sub-Saharan Africa is home to only 12% of the world's population but has over 70% of the global burden of HIV.^1^ The outlook of HIV outcomes on the continent has greatly improved over the past two decades, largely due to the massive rollout of anti-retroviral drugs (ARVs).^2^ Ghana, a west African country, has a steadily declining prevalence of 1.7% in 2019 from 2.0% in 2014.^3^

Anxiety disorders are characterised by an excessive or irrational fear response, related cognitive and behavioural responses, and clinically significant distress and dysfunction.^4^ It has been consistently shown that these disorders have a higher prevalence in PLWHA than in the general population.^5^–^7^

The estimated global prevalence of anxiety among PLWHA is 27.9%, ranging between 1% and 47.8%. 8 Studies from African countries (other than Ghana) report prevalence rates within this range. 5,6,9,10 Ghanaian studies have consistently recorded much higher prevalence rates for anxiety in PLWHA. Studies conducted in Accra, Kumasi and Cape Coast reported a prevalence of 75.3%, 78% and 46.6%, respectively.^11^–^13^

Anxiety has been strongly linked to risky sexual practices, delayed presentation for treatment and poor adherence to anti-retroviral therapy, all of which have adverse effects on the health of both PLWHA and the general public.^14^–^16^

As Ghana has missed the ambitious target set by the Joint United Nations Programme on HIV/AIDS (UNAIDS) ‘90-90-90’ to attain 90% HIV-status awareness, 90% sustained ART for all diagnosed with HIV and 90% viral suppression for all PLWHA on ARVs by 2020, it may be worthwhile to explore less obvious, indirect obstacles to achieving the desired control over the HIV/AIDS pandemic, such as anxiety and other mental illnesses in PLWHA.^17^ Our study sought to assess and update information on the prevalence of anxiety and its associated factors among HIV patients attending a large tertiary medical centre in Ghana.

## Methods

### Study Design & population

This was a cross-sectional analysis of data from a larger study.^18^ The study was designed as a case-control study to assess the cardiovascular risk among PLWHA. The study sample included PLWHA on cART compared with age- and sex-matched PLWHA who are cART naïve and HIV-negative controls. The current analysis is limited to PLWHA aged ≥30 years (a criterion for inclusion into the original study) receiving cART for at least one year and PLWHA who were cART naïve. The study was conducted at the HIV clinic of a tertiary medical facility in Ghana. Ethical approval for the study was obtained from Kwame Nkrumah University of Science and Technology Committee of Human Research Publications and Ethics (CHRPE/AP/281/16). All participants provided written informed consent.

### Measures

Standard data collection questionnaires were administered to all 495 PLWHA to collect demographic information, including age, sex, educational status and location of residence (rural, peri-urban and urban). Clinical data such as CD4 count, viral load and duration since initial positive HIV test were collected by interview and review of medical records. HADS was administered to assess for anxiety and depression. Anxiety-positive cases were defined by a HADS Anxiety sub-scale (HADS-A) score of 8 or more, with mild (8–10), moderate (11–14) and severe (15–21) cases defined. The same definitions were applied to depression cases using the HADS Depression sub-scale (HADS-D).

### Statistical analysis

Data for the study were collected directly into a REDCap database, cleaned daily for abnormal values and observations and transferred to R statistical software for analysis. 19–21 Anxiety was first derived as absent, mild, moderate or severe based on the HADS-A score. This was further categorised as present or absent.

The prevalence of anxiety was determined and reported with its 95% binomial exact confidence intervals. The relationship between the presence of anxiety and other covariates was initially tabulated and presented with the corresponding p-values derived from either a student's t-test if continuous or a chi-square test if categorical. Association between clinical and epidemiological characteristics and the presence of anxiety was then explored in a logistic regression. The relationships were expressed as odd ratios, their 95% confidence interval and likelihood ratio p-values. The covariates determined to be significantly associated with anxiety in the crude analysis were included in a multivariate regression model to determine covariates independently associated with anxiety. For all analyses, a p-value less than 0.05 was considered statistically significant.

## Results

### Characteristics of Study Participants

The study sample comprised 495 PLWHA with a mean age of 44.1 ± 9.05 years and predominantly females (75.8%). Two hundred and forty (48.5%) were cART naïve at enrolment into the study, and the remainder (51.5%) were on cART.

### Prevalence of Anxiety

The overall prevalence of anxiety was 61.0 % (95%CI: 56.6 – 65.3). The proportions of respondents with mild, moderate, and severe anxiety were 22.6%, 31.3% and 7.1%, respectively. Among those with diagnosis of HIV less for than 6 months, the prevalence of anxiety was 64.3% vs 59.1% among those with diagnosis more than 6 months. (Table 1)

**Table 1:** Prevalence of anxiety among Ghanaians living with HIV

	Time since HIV Diagnosis					
	<6months (n= 182)		>=6 months (n=313)		Total (n=495)	
Anxiety Level	n	% (95%CI)	N	% (95%CI)	n	% (95%CI)
**None**	65	35.7 (29.0–43.0)	128	40.9 (35.6 - 46.5)	193	39 (34.8 - 43.4)
**Anxiety Present**	117	64.3 (57.0 – 71.0)	185	59.1 (53.5 - 64.4)	302	61.0 (56.6 - 65.2)
**Mild**	34	18.7 (13.6 - 25.1)	78	24.9 (20.4 – 30.0)	112	22.6 (19.1 - 26.5)
**Moderate**	68	37.4 (30.6 - 44.7)	87	27.8 (23.1 – 33.0)	155	31.3 (27.4 - 35.5)
**Severe**	15	8.2 (5.0 - 13.3)	20	6.4 (4.2 - 9.7)	35	7.1 (5.1 - 9.7)

CI, confidence interval

### Comparison of demographic and clinical characteristics according to Anxiety status

Table 2 shows a comparison of demographic and clinical characteristics of study participants according to anxiety status. There were no significant differences in mean age, sex and educational attainment between the two groups. Compared with those without anxiety, PLWHA with anxiety were more likely to dwell in urban residence (66.3% vs. 76.8%), to be current user of alcohol (7.8% vs. 9.1%) and more likely to have depression. There were also differences in employment status between the two groups. Of note, severity of clinical status, viral load and CD4 counts were not significantly different between the two groups, as well as cART exposure status.

**Table 2:** Demographic and clinical characteristics of study participants according to anxiety status

Characteristics	No Anxiety Present (n=193)	Anxiety Present (n=302)	Total (n=495)	P-value
	n (%)	n (%)	n (%)	
**Age, mean ± SD**	44.8(9.95)	43.7 (8.42)	44.1 (9.05)	0.187
**Female, n (%)**	142(73.58)	233 (77.15)	375 (75.76)	
**Educational level**				0.166
**None**	41(21.24)	86 (28.48)	127 (25.66)	
**Primary**	72(37.31)	99 (32.78)	171 (34.55)	
**Secondary**	70(36.27)	94 (31.13)	164 (33.13)	
**tertiary**	10(5.18)	23 (7.62)	33 (6.67)	
**Location of residence**				0.036
**Rural**	10 (5.18)	12 (3.97)	22 (4.44)	
**Semi-urban**	55 (28.5)	58 (19.21)	113 (22.83)	
**urban**	128 (66.32)	232 (76.82)	360 (72.73)	
**Employment**				< 0.001
**Self-employed**	139 (72.02)	232 (76.82)	371 (74.95)	
**Unemployed**	29 (15.03)	31 (10.26)	60 (12.12)	
**Private company employee**	9 (4.66)	32 (10.6)	41 (8.28)	
**Government employee**	8 (4.15)	0 (0)	8 (1.62)	
**Homemaker**	3 (1.55)	5 (1.66)	8 (1.62)	
**Retired**	5 (2.59)	2 (0.66)	7 (1.41)	
**Marital status**				0.446
**Single**	97 (50.26)	157 (51.99)	254 (51.31)	
**Married**	24 (12.44)	30 (9.93)	54 (10.91)	
**Widowed**	39 (20.21)	57 (18.87)	96 (19.39)	
**separated**	14 (7.25)	15 (4.97)	29 (5.86)	
**divorced**	19 (9.84)	43 (14.24)	62 (12.53)	
**Monthly income**				0.446
**>1000 GHS**	30 (15.54)	51 (16.89)	81 (16.36)	
**500–1000 GHS**	60 (31.09)	96 (31.79)	156 (31.52)	
**100–500 GHS**	63 (32.64)	102 (33.77)	165 (33.33)	
**< 100 GHS**	12 (6.22)	25 (8.28)	37 (7.47)	
**Don't Know**	28 (14.51)	28 (9.27)	56 (11.31)	
**Duration of HIV diagnosis**				0.255
**< 6months**	65 (33.68)	117 (38.74)	182 (36.77)	
**>= 6 months**	128 (66.32)	185 (61.26)	313 (63.23)	
**WHO clinical stage at diagnosis**				0.179
**1 & 2**	157 (81.35)	224 (74.17)	381 (76.97)	
**3 & 4**	34 (17.62)	73 (24.17)	107 (21.62)	
**No data**	2 (1.04)	5 (1.66)	7 (1.41)	
**Alcohol use**				0.035
**Current**	15 (7.77)	30 (9.93)	45 (9.09)	
**Former**	58 (30.05)	120 (39.74)	178 (35.96)	
**never**	120 (62.18)	152 (50.33)	272 (54.95)	
**Cigarette use**				0.871
**Current**	3 (1.55)	4 (1.32)	7 (1.41)	
**Former**	10 (5.18)	19 (6.29)	29 (5.86)	
**never**	180 (93.26)	279 (92.38)	459 (92.73)	
**Suppressed viral load**				0.332
**Suppressed**	93 (48.19)	125 (41.39)	218 (44.04)	
**Unsuppressed**	95 (49.22)	168 (55.63)	263 (53.13)	
**Not done**	5 (2.59)	9 (2.98)	14 (2.83)	
**Depression, n (%)**				< 0.001
**Normal**	147 (76.17)	73 (24.17)	220 (44.44)	
**Borderline**	24 (12.44)	83 (27.48)	107 (21.62)	
**Abnormal**	22 (11.4)	146 (48.34)	168 (33.94)	
**cART status n(%)**				0.225
**cART**	106 (54.92)	149 (49.34)	255 (51.52)	
**Naïve**	87 (45.08)	153 (50.66)	240 (48.48)	
**CD4 count, current, mean ± SD**	512 (350)	467 (337)	484 (343)	0.160
**cART, combination anti-retroviral therapy**				

### Factors Associated with Anxiety among PLWHA

Unadjusted analysis identified four potential factors namely urban residence, being unemployed, alcohol use and depression as potential factors associated with anxiety.

Adjusting for other significant covariates however, urban place of residence (aOR: 1.67, 95%CI: 1.12, - 2.51), alcohol use (aOR: 1.64, 95%CI: 1.13 - 2.38), borderline depression (OR=6.43, 95%CI: 3.73 - 11.06) and depression (aOR: 13.62, 95%CI: 7.91 - 23.45) were significantly associated with the odds of having anxiety in our study population.

## Discussion

We found an overall prevalence of 61.0% for anxiety in patients with HIV, without observing any significant difference between patients with recently vs. established diagnosis of HIV. Brandt et al calculated a median prevalence of 27.9% from a range of 1.0% to 47.8% in a 2017 review of publications reporting on anxiety in PLWHA.^8^ Regarding prevalence, two trends were observed in their study which bear mentioning. Firstly, questionnaire-based assessments yielded significantly higher median prevalence of 33.3% compared with diagnostic interviews with a median rate of 22.9%. Secondly, anxiety prevalence rates were higher in developed countries (median=28.5%) than in developing countries (median= 22.9%).

Recent findings from studies conducted in Ghana, which were not included in Brandt's review, appear to report somewhat higher prevalence rates of anxiety in PLWHA in Ghana. For instance, a 2018 study in PLWHA receiving HAART from a tertiary hospital in Kumasi reported a high prevalence of 78.0% using the Depression Anxiety and Stress Scale (DASS-21). 12 Elsewhere in the Central region of Ghana, Siakwa et al reported 46.6% using the Mini International Neuropsychiatric Interview (MINI). 13 It is noteworthy that in females, who constituted 58.0% of their sample, the prevalence of anxiety disorders was 72.0%. In Accra, Asante et al recorded a prevalence of 75.0% using the DASS-42 in PLWHA receiving care at Korle Bu Teaching Hospital. In contrast, one study in a similar setting in Ado-Ekiti, in Nigeria, reported a prevalence of 32.6% with HADS, while another conducted in Lagos, Nigeria, found a prevalence of anxiety disorders of 21.7% in HIV-clinic attendees using Schedule for Clinical Assessment in Neuropsychiatry (SCAN). 6,22

There are several factors which could possibly account for this high prevalence observed in our study. One of these is the peculiarities of the major local language, Akan-Twi into which the questionnaire had to be translated for respondents with low educational attainment. Another factor could be the possibility of particularly high levels of illness anxiety in Ghanaians. An example of this is seen in a 2017 study of breast cancer patients in Accra which found a 92.5% prevalence of anxiety using HADS whereas a 2020 systematic review estimated the prevalence of anxiety in breast cancer patients to be 41.9%. 23,24 This is an area for further study.

There is abundant evidence of a higher prevalence of anxiety in urban areas in comparison to rural areas but this trend may not necessarily be the same in all PLWHA. 25,26 Unfortunately, not many studies have addressed this directly. A couple of studies from the USA however compared other related mental health parameters in rural and urban settings. One study found similar levels of AIDS-related stress in men in rural and urban settings, while another found higher levels of psychological distress in rural-dwelling PLWHA. 27,28 It should however be noted that in the former, about half of the sample were homosexual men and in the former, 32% listed homosexual contact as the mode of infection in the latter. Our study may be the first to identify increased anxiety in urban-dwelling PLWHA in sub-Saharan Africa. The demographic differences must be taken into consideration when comparing results in Africa to those in the USA and other western countries because homosexuals experience more stigma in rural areas unrelated to HIV status. 29 Notwithstanding, rural dwelling and lower education attainment have been universally linked to increased stigma against PLWHA, an important contributor to anxiety, depression and mental distress in this group. 30–32 It would appear that the effect of higher stigma may not be as strong as that of other non-HIV related, anxiogenic environmental factors such as higher stress levels, noise pollution and occupational stress which are worse in urban areas. Also, rural dwellers are likely to have a closer-knit social network which could prove protective against anxiety.

The association found between anxiety and alcohol use is most likely a reflection of the widely observed trend where people with significant anxiety are more likely to use alcohol for its anxiolytic effect. 33 This is nonetheless an important finding because alcohol abuse adds on multiple complications with grave implications for the individual and public health. Alcohol abuse may further lower immunity in PLWHA leading to a poorer treatment response. 34 There is also the problem of impaired judgement with alcohol intoxication which may lead to increased likelihood of engaging in unprotected sex, putting one at risk of contracting and spreading HIV and other sexually transmitted diseases. 35

We also identified a strong association between anxiety and depression. This observation is not a peculiar one as these two conditions have been reported to occur co-morbidly over 50% of the time. 36 It is however noteworthy, given the high prevalence rates reported in this and other studies of anxiety and depression in PLWHA, because of the negative effects of comorbidity such as higher chronicity and treatment resistance, slower recovery and greater disability. The outlook is bound to be even worse when the individual effects of these disorders in PLWHA are combined.

Our findings indicate that Ghanaian PLWHA may have a peculiarly high burden of anxiety and possibly mental illness in general. Knowing the adverse effects of anxiety on adherence to medication, safe sexual practices and other important aspects of individual and public health care as regards PLWHA a closer look must be taken into the mental health aspects of HIV care. In order to achieve our goals of stemming the HIV pandemic as a nation, more research and policy changes must be undertaken to facilitate the understanding and management of anxiety, depression and other mental disorders in PLWHA. Screening for common mental disorders should be integrated as part of routine care.

This study employed a cross-sectional design which limits our ability to draw any causal inferences. Also, findings from a single site study may not be generalizable to other parts of the country and continent. We also employed a questionnaire-based tool which has been shown to give higher prevalence rates than the gold standard and other interview-based assessments. 8 Our results are nonetheless similar to previous studies carried out in PLWHA attending OPD clinics in other parts of Ghana.

## Conclusion

Six (6) out of every 10 PLWHA in this Ghanaian sample had evidence of anxiety, which was independently associated with urban dwelling, alcohol use and depression. Routine screening for anxiety disorders, and other common mental disorders, as well as building treatment capacity referral networks is pivotal to comprehensive care of PLWHA. More liaison with local mental health services and research into the reasons for the high prevalence of anxiety is needed. Further research also needs to be carried out to explore the contributors to, consequences and costs of anxiety and other mental illnesses in PLWHA.

## Figures and Tables

**Table 3:** Multivariate logistic analysis of risk factors for anxiety for all patients

Characteristics	Unadjusted OR (95%CI)	p-value	Adjusted* OR (95% CI)	p-value
**Age**	0.87 (0.71,1.06)	0.171		
**Sex, Female**	1.21 (0.8,1.84)	0.367		
**Educational level**		0.160		
**None**	Ref			
**Primary**	0.66 (0.41,1.06)			
**Secondary**	0.64 (0.39,1.04)			
**Tertiary**	1.1 (0.48,2.52)			
**Urban residence**	1.68 (1.13,2.51)	0.011	1.67 (1.12,2.51)	0.013
**Unemployed**	0.61 (0.37,0.99)	0.048	0.62 (0.37,1.02)	0.058
**Marital status**		0.442		
**Married**	Ref			
**Single**	0.77 (0.43,1.4)			
**Widowed**	0.9 (0.56,1.46)			
**separated**	0.66 (0.31,1.43)			
**divorced**	1.4 (0.77,2.54)			
**Monthly income**		0.454		
**>1000 GHC**	Ref			
**500–1000 GHc**	0.94 (0.54,1.64)			
**100–500 GHC**	0.95 (0.55,1.65)			
**< 100 GHC**	1.23 (0.54,2.79)			
**Dont Know**	0.59 (0.29,1.17)			
**Possession of Insurance**		0.282		
**Valid**	Ref			
**Not Valid**	0.74 (0.46,1.2)			
**None**	1.25 (0.72,2.18)			
**HIV diag. Duration, >= 6 months**	0.8 (0.55,1.17)	0.253		
**WHO clinical stage at diagnosis**		0.172		
**1 & 2**	Ref			
**3 & 4**	1.5 (0.95,2.37)			
**No data**	1.75 (0.34,9.15)			
**Alcohol use, Yes**	1.62 (1.12,2.34)	0.010	1.64 (1.13,2.38)	0.009
**Cigarette use**		0.859		
**Current**	Ref			
**Former**	1.42 (0.27,7.66)			
**never**	1.16 (0.26,5.26)			
**Suppressed viral load**		0.332		
**Suppressed**	Ref			
**Unsuppressed**	1.32 (0.91,1.9)			
**Not done**	1.34 (0.43,4.13)			
**Depression**		< 0.001		< 0.001
**Normal**	Ref		Ref	
**Borderline**	6.96 (4.08,11.88)		6.43 (3.73,11.06)	
**Abnormal**	13.36 (7.87,22.68)		13.62 (7.91,23.45)	
**ART Status, Naïve**	1.25 (0.87,1.8)	0.225		
**CD4 count, current**	0.96 (0.91,1.01)	0.157		

OR, odds ratio; CI, confidence interval

* adjusted for depression, employment status, residence and alcohol use.
